# Regulatory T-cell remodeling across the HBV infection–cirrhosis continuum

**DOI:** 10.3389/fimmu.2026.1905693

**Published:** 2026-09-03

**Authors:** Yudan Fu, Yi Wang, Jiqi Ouyang, Wenliang Lv

**Affiliations:** 1 Department of Infection, Guang’anmen Hospital, China Academy of Chinese Medical Sciences, Beijing, China; 2 Graduate School, Beijing University of Chinese Medicine, Beijing, China

**Keywords:** hepatitis B virus, immune regulation, liver cirrhosis, liver fibrosis, regulatory T cells, Treg/Th17 imbalance

## Abstract

Regulatory T cells (Tregs) have varied functions in the course of hepatitis B virus (HBV) infection and cirrhosis. Regulatory T cells (Tregs) generally restrict excessive immune-mediated liver injury in acute HBV infection but may also inhibit antiviral immunity. During chronic infection, expanded and intrahepatically enriched Tregs suppress HBV-specific effector T-cell responses, maintain antiviral immune hyporesponsiveness and viral persistence, and limit chronic necroinflammatory injury. As the extent of HBV-related liver fibrosis progresses, there is a relative deficiency in both Tregs and Th17 cells. Treg cells are not only inhibitory to fibrosis; they are also interconnected with the immune-fibrotic microenvironment established by HSCs, and inflammation, HSC activation, extracellular matrix deposition and Treg/Th17 imbalance are all interrelated. Tregs in HBV-associated cirrhosis are highly heterogeneous, compartmentally distributed, functionally reprogrammed, and have joined an intrahepatic immunosuppressive-fibrotic network that includes myeloid cells, HSCs, natural killer cells, CD8+ T cells and Th17 cells. This paper will introduce modifications in Treg abundance, phenotype, distribution and function during the progressive alteration of these periods, as well as microenvironmental factors, Treg/Th17 dysregulation and specific therapeutic options. Therefore, the effect of regulation of Treg activity in HBV-associated liver disease is context-dependent and stage-specific, not uniform enhancement or suppression.

## 1 Introduction

Hepatitis B virus (HBV) is a serious infectious disease in many parts of the world that is a cause of both liver cirrhosis and hepatocellular carcinoma (HCC) (1). According to the latest WHO report, about 254 million people worldwide were living with chronic HBV infection in 2022, and around 1.2 million new cases were occurring each year; most of the related deaths were caused by cirrhosis and HCC (2). More than 80% of HCC cases are due to HBV infection; thus, these patients have shorter survival and poorer quality of life and pose a significant threat to public health (3). China has the highest burden of hepatitis B in the world, with about 75 million people living with chronic HBV infection and accounting for almost one-third of the global HBV-infected population; thus, HBV-related cirrhosis and HCC are among the most serious diseases in China today (4).

The course of natural history for HBV-related liver disease is a gradual progression from chronic hepatitis to liver fibrosis and cirrhosis, thus substantially increasing the risk of HCC. Cirrhosis is generally the result of long-term damage to hepatocytes and impaired tissue repair due to dysregulated host immunity in chronic hepatitis B infection, rather than direct cytotoxicity by HBV. Liver inflammation, fibrosis and subsequent irreversible architectural damage are generally the result of extended dysregulation of the host’s adaptive and innate immune systems. Therefore, the course of HBV infection-to-cirrhosis can be considered a dynamic pathological process driven by viral persistence, immune-mediated injury and aberrant tissue repair. Thus, research on immune-regulatory mechanisms is now being conducted to explore how cirrhosis develops and how to develop immune-targeted drugs.

Regulatory T cells (Tregs) are an immunosuppressive type of T cell that is typically CD4^+^ CD25^+^ forkhead box P3 (FOXP3)^+^. They suppress effector T-cell activation and inflammatory spread in multiple ways, such as releasing inhibitory cytokines such as interleukin-10 (IL-10) and transforming growth factor-beta (TGF-β), expressing inhibitory molecules such as cytotoxic T-lymphocyte-associated protein 4 (CTLA-4) and programmed cell death protein 1 (PD-1), and engaging in contact-dependent cell interactions. During the course of HBV-associated liver disease, Tregs have been identified as key regulators of the balance between antiviral clearance and immune-mediated damage to hepatocytes, and they exert protective effects by restraining excessive immune activation and thus reducing immune-induced damage to hepatocytes. At the same time, continuous suppression of HBV-specific effector T-cells may be detrimental to the host and thus promote the prolonged persistence of HBV through impaired antiviral immunity.

The two modes are not fixed; rather, they exhibit strong stage-specificity and functional plasticity as a result of disease progression, remodelling of the intrahepatic microenvironment, and alterations in the Treg/Th17 ratio. As chronic hepatitis B infection progresses, extended exposure to antigens has led to the accumulation of inflammatory cytokines, HSC activation, alterations in the extracellular matrix (ECM), changes in immune cells, and thus formed a dynamic pathological microenvironment in the liver. The Microenvironment constantly modifies the recruitment, phenotypic stability and suppressive function of Tregs.

**Figure 1:**
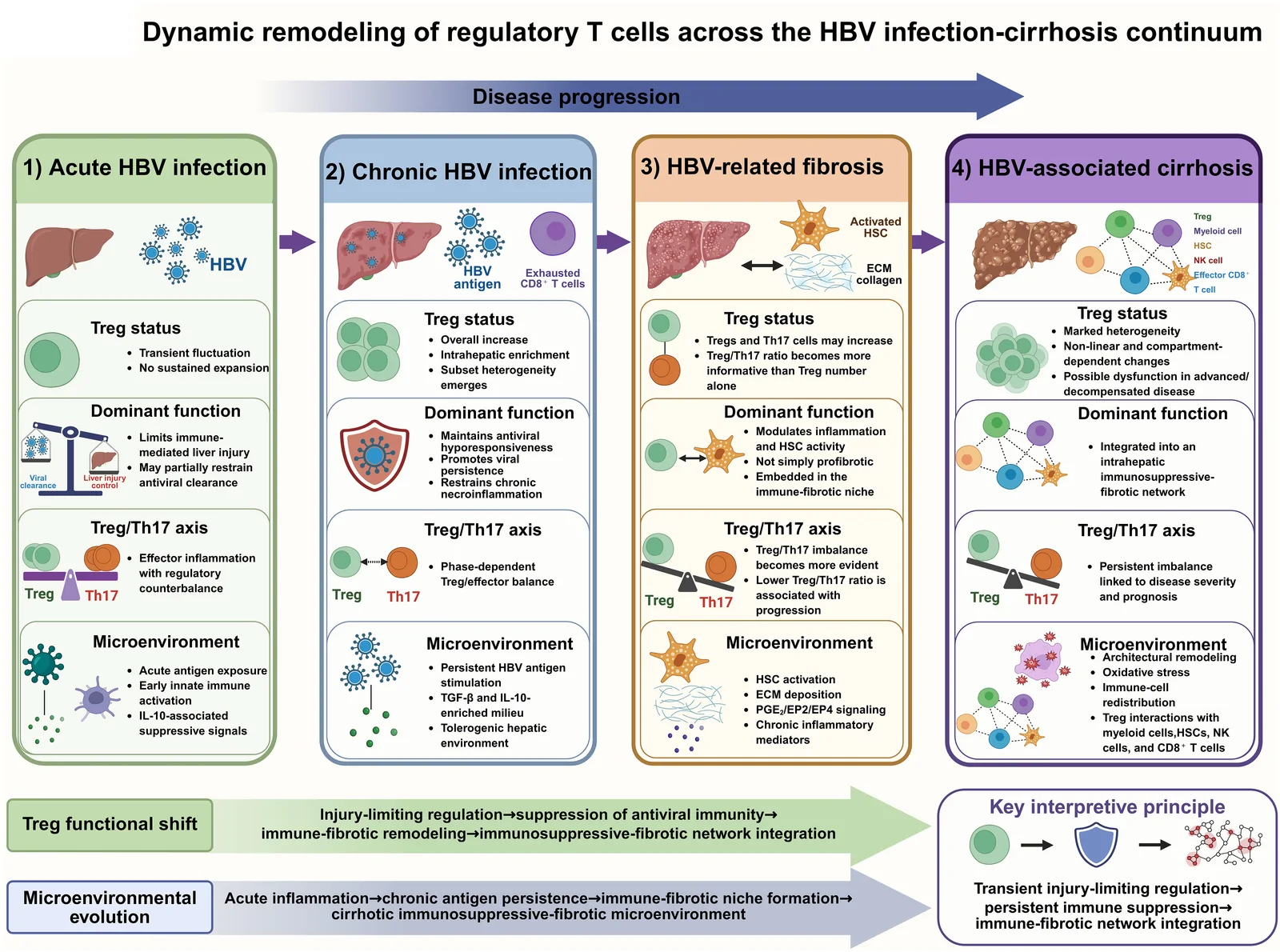
Dynamic remodeling of regulatory T cells across the HBV infection–cirrhosis continuum. The schematic summarizes stage-dependent changes in regulatory T-cell (Treg) abundance, phenotype, localization, and function from acute HBV infection through chronic infection and liver fibrosis to cirrhosis. Each stage is organized according to Treg status, dominant function, the Treg/Th17 axis, and major microenvironmental influences. Overall, Tregs shift from transient limitation of immune-mediated injury to persistent suppression of antiviral immunity and integration into an intrahepatic immunosuppressive–fibrotic network. Their functional effects are determined by disease stage, the Treg/Th17 balance, and the surrounding hepatic microenvironment. CD8^+^ T cell, cluster of differentiation 8-positive T cell; ECM, extracellular matrix; HBV, hepatitis B virus; HSC, hepatic stellate cell; NK cell, natural killer cell; Treg, regulatory T cell. Created with BioRender.com.

In summary, Tregs in HBV-related liver disease are not static immunosuppressive cells, but rather key regulatory hubs that undergo continuous functional remodeling as the disease progresses and the intrahepatic microenvironment evolves. This review centers on the progression from HBV infection to liver cirrhosis and systematically summarizes the dynamic changes in the abundance, phenotype, and function of Tregs across four stages: acute infection, chronic infection, liver fibrosis, and cirrhosis. We focus on their stage-dependent dual functional characteristics and the mechanisms by which the intrahepatic microenvironment drives Treg functional remodeling, and further discuss the significance of Treg/Th17 imbalance in disease progression and its potential value as a therapeutic target. The stage-dependent remodeling of Tregs across the HBV infection–cirrhosis continuum is summarized in **Figure 1**.

## 2 Stage-dependent dynamics and functional features of Tregs in the progression from HBV infection to cirrhosis

Changes in Tregs across HBV infection and HBV-associated liver disease progression are characterized by marked stage-specificity and functional reprogramming.In acute HBV infection and early chronic hepatitis, Tregs primarily serve to modulate the equilibrium between antiviral effector immune responses and immunological liver damage. With progression from chronic hepatic inflammation to fibrosis and cirrhosis, Tregs increasingly transcend their conventional role in immune regulation and emerge as multifunctional contributors to the construction of a profibrotic microenvironment, the formation of local immunosuppressive circuits, and the perpetuation of disease progression. The key to understanding this process is not to determine a unidirectional change in Treg abundance, but to delineate the dominant functions that Tregs assume at different disease stages and their reciprocal shaping relationship with the intrahepatic microenvironment.

### 2.1 Time-dependent dynamics and protective immunoregulatory functions of Tregs in acute HBV infection

The main immunological problem in acute hepatitis B (AHB) is how to rapidly generate a strong antiviral immune response that can clear HBV without causing harm to the liver as a result of excessive immune activation. Among immunologically competent adults, acute infection generally follows a self-limited course, and viral clearance is mainly achieved by functional HBV-specific CD4+ and CD8+ T cells. However, liver cell injury is also primarily caused by immune-mediated effector mechanisms rather than direct cytotoxicity from HBV. Tregs help to regulate the balance between HBV elimination and the extent of liver injury in this process. Suppression of effector T-cell activation may reduce immune-mediated liver injury but may also weaken the capacity for antiviral defence.

Current evidence suggests that Treg alterations during acute HBV infection do not follow a pattern of sustained increase or unidirectional decline, but instead exhibit temporal fluctuations and a dynamic regulatory pattern coupled with the inflammatory process. Previous studies reported that patients with AHB showed peripheral CD4^+^ CD25^hi Treg frequencies similar to those of healthy controls, without persistent Treg expansion (5). Instead, the more prominent immunological feature at this stage was an enhanced Th17-related effector inflammatory response (5). Liang et al. further reported that peripheral Th17 cells and IL-17A levels were significantly increased in patients with AHB. Although FOXP3^+^ Tregs could also increase in parallel, the Treg/Th17 ratio did not differ significantly from that in healthy controls (6). These findings suggest that the immune profile of acute HBV infection is better characterized by a predominance of effector inflammatory responses accompanied by regulatory counterbalance, rather than by the establishment of a stable Treg-dominated immunosuppressive milieu.

Longitudinal dynamic observations have further revealed the stage-specific remodeling characteristics of Tregs in AHB.Xu et al. reported that, in patients with AHB, circulating peripheral Tregs remained at relatively low levels during the early phase of disease onset, then showed a transient increase during the recovery phase, and returned to normal levels after complete disease resolution (7). A separate study on the dynamic immune response in AHB demonstrated that, relative to healthy controls, both Th17 cells and Tregs were elevated during the acute phase (8). Upon transition to the early convalescent phase, Th17 cells decreased while Tregs continued to rise, leading to a decline in the Th17/Treg ratio. During complete recovery, Treg levels gradually subsided but could still remain modestly above those of healthy controls, whereas the Th17/Treg ratio gradually approached the normal range. This study further showed that, in the acute phase of AHB, HBsAg and HBeAg levels were positively associated with the Th17/Treg ratio, indicating that viral antigen load may participate in determining the pattern of immune equilibrium at this stage.

At the functional level, the principal role of Tregs in AHB appears to be the limitation of immune-mediated liver injury, although this protective effect may come at the cost of partially reduced antiviral clearance efficiency. Animal experiments suggest that depletion of Foxp3^+^ Tregs in an acute HBV infection model enhances early immune control and promotes the clearance of hepatitis B e antigen (HBeAg) and hepatitis B surface antigen (HBsAg), but simultaneously aggravates hepatic immunopathological injury (9). This study also indicates that Tregs suppress not only HBV-specific adaptive immunity but also innate immune responses during the early phase of infection (9). Dunn et al., in a temporal analysis of early immune responses during acute HBV infection, found that the viremic phase was accompanied by increased IL-10 levels, coinciding with transient suppression of natural killer (NK)-cell and T-cell responses (10). Although this phenomenon cannot be directly equated with Treg expansion, it suggests the presence of an immunosuppressive microenvironment during the acute phase that is functionally consistent with Treg activity.

Collectively, the present evidence suggests that Treg alterations during AHB should be viewed as a time-varying regulatory process. Tregs regulate the antiviral effector response in the liver to prevent immunopathological damage, but this balance is fragile and unstable; if they are too suppressive or prolonged, the HBV-specific effector immune response will be weakened and timely viral clearance will be delayed. The idea of this view will provide the foundation for building immune-surveillance and early-treatment systems for acute hepatitis B virus (HBV) infection.

### 2.2 Stage-dependent heterogeneity and functional duality of Tregs in chronic HBV infection

The immunological essence of chronic HBV infection is a long-term dynamic coexistence between the virus and the host immune system under continuous immune surveillance. It is characterized by transitions among multiple immunological phases, including the immune-tolerant phase, immune-active phase or chronic active hepatitis phase, low-replicative phase or immune-control phase, and reactivation phase. Across these immunological phases, Tregs show substantial differences in number, subset architecture, tissue localization, and functional state. In general, patients with chronic HBV infection exhibit an overall expansion of Tregs in the peripheral circulation and within the liver, yet this expansion is heterogeneous and characterized by clear phase-dependent diversity and functional adaptability.

In the immune-tolerant or high-replicative state, the core function of Tregs is to maintain antiviral immune hyporesponsiveness against HBV, thereby promoting viral persistence.Several studies have shown that, during the immune-tolerant phase, the frequency of circulating CD4^+^ CD25^+^ FOXP3^+^ Tregs is markedly increased compared with healthy controls and subjects with spontaneous HBV resolution. Moreover, Treg frequency correlates positively with serum HBV DNA levels and negatively with anti-HBe antibody status (5, 7). Using major histocompatibility complex (MHC) class II tetramer technology, Tsai et al. identified a high frequency of hepatitis B core antigen (HBcAg)-specific CD4^+^ CD25^+^ Tregs in patients in the immune-tolerant phase, with FOXP3^+^ expression as their predominant feature (11). After Treg depletion, the interferon-γ (IFN-γ) response of peripheral blood mononuclear cells (PBMCs) to HBV antigen stimulation was significantly restored, indicating that Tregs constitute an important mechanism maintaining T-cell hyporesponsiveness during immune tolerance (12).

After progression into the immune activation phase, changes in Tregs are more strongly associated with the intensity of hepatic inflammation and exhibit selective remodeling of specific subsets, most notably the preferential expansion of memory Tregs and their increased accumulation within the liver (13). Cheng et al. further classified FOXP3^+^ Tregs into resting Tregs (rTregs, CD45RA^+^FOXP3^lo) and memory Tregs (mTregs, CD45RA⁻FOXP3^hi). They found that mTregs were selectively increased during the inflammatory phase of chronic HBV infection and were positively correlated with serum alanine aminotransferase (ALT) levels, suggesting that activation of memory Tregs is closely associated with chronic inflammatory activity (13). During the relatively low-replicative or quiescent phase, peripheral Treg levels may partially decline, but they do not necessarily return completely to the levels observed in healthy individuals (5). In some patients, peripheral Treg levels remain higher than those in individuals who have fully recovered with HBsAg clearance, suggesting that the persistence of low-level covalently closed circular DNA (cccDNA) may still exert some influence on Treg homeostasis (7).

Studies suggest that, in patients with chronic HBV infection, the frequency of intrahepatic FOXP3^+^ Tregs is generally higher than that in peripheral blood and is associated with the degree of hepatic inflammation (7). Within the liver, locally enriched HBV antigens, a cytokine milieu involving TGF-β and IL-10, and modulation by innate immune cells such as Kupffer cells may collectively enhance Treg expansion and suppressive activity, giving rise to an immunoregulatory landscape that differs from that observed in the peripheral circulation. Therefore, when evaluating the clinical significance of Tregs during chronic HBV infection, it is necessary to distinguish the distinct immune compartmental states reflected by different sample sources.

In terms of functional duality, Tregs in chronic HBV infection simultaneously mediate two opposing biological consequences. On the one hand, Tregs help attenuate immune-mediated hepatocellular injury and limit excessive progression of chronic necroinflammation by suppressing excessive effector T-cell activation and amplification of inflammatory cascades. This protective role is particularly important during the immune-active phase, because in the absence of effective regulatory counterbalance, persistent HBV-specific effector T-cell activation may lead to more severe liver tissue damage. On the other hand, Tregs weaken effective antiviral immunity and promote viral persistence and maintenance of chronic infection by suppressing the proliferation, cytokine secretion, and cytotoxic activity of HBV-specific CD4^+^ and CD8^+^ T cells.Particularly in the immune-tolerant or high-viremic state, sustained enhancement of Treg-mediated suppression is more likely to consolidate HBV-related antiviral immune hyporesponsiveness. During the immune-active phase, this suppressive effect does not disappear; rather, it enters into a more complex dynamic interplay with effector immunity, placing the host in a persistent paradoxical state in which limitation of liver injury coexists with impaired viral clearance.

With the persistence of chronic infection, regulatory T cells (Tregs) have changed their function from “transient suppression of inflammation in the acute stage” to “central regulators of viral persistence and prolonged inflammation”; at this time, local immune reprogramming occurs that promotes hepatic fibrosis and cirrhosis.

### 2.3 Dynamic alterations in Tregs during HBV-related liver fibrosis and their association with the fibrotic microenvironment

HBV-related liver fibrosis is the result of continuous stimulation of chronic viral antigen, repeated immune-mediated liver damage and abnormal tissue repair. The primary pathological features are prolonged activation of hepatic stellate cells (HSCs) and abnormal deposition of extracellular matrix. Hepatic fibrosis at this time is still regarded as a reversible pathological process, but if left untreated, it will gradually develop into irreversible structural changes of liver cirrhosis. At this time, the role of Tregs has been extended beyond their previous function as regulators of the immune system in chronic infection to inhibit inflammation and maintain antiviral hyporesponsiveness. They gradually become integrated into the profibrotic microenvironment and actively regulate HSC behaviour and the process of fibrosis.

Available evidence indicates that both circulating Tregs and Th17 cells can be elevated in HBV-associated hepatic fibrosis. Nevertheless, the Treg/Th17 ratio, rather than the absolute abundance of Tregs alone, may have greater relevance for evaluating the progression of fibrosis. The Treg/Th17 ratio declines as hepatic inflammation becomes more severe, ALT levels rise, and fibrosis progresses, indicating that the critical feature of HBV-associated hepatic fibrosis is not merely Treg expansion, but disruption of the immunological equilibrium between Tregs and Th17 cells (14–16).

Li et al. further revealed the bidirectional nature of Treg dynamics at this stage (14). The frequency of peripheral Tregs was positively correlated with HBV DNA load, but negatively correlated with the degree of liver injury and fibrosis stage. Correspondingly, Th17-cell frequency was positively associated with the severity of liver injury and fibrosis, but showed no significant correlation with HBV DNA load (14). *In vitro* co-culture experiments showed that CD4^+^ CD25^+^ cells derived from patients with chronic hepatitis B dose-dependently inhibited HSC proliferation and activation, whereas IL-17 promoted HSC proliferation and the acquisition of a profibrotic phenotype. In supportive animal experiments, depletion of CD25^+^cells aggravated liver injury and fibrosis, whereas depletion of IL-17^+^cells attenuated fibrosis (14). These findings suggest that Tregs are not unidirectional profibrotic effector cells in HBV-related liver fibrosis. Their suppression of inflammation and HSC activation may still confer a degree of protection; however, when Tregs are relatively insufficient or the Treg/Th17 ratio becomes imbalanced, inflammatory amplification and fibrotic progression may be exacerbated (14, 17). It should be noted that the conclusions drawn from these *in vitro* and animal experiments require further validation in human studies, and the relevant causal relationships should still be interpreted with caution.

Other studies have shown that the functions of Tregs in HBV-induced liver fibrosis are regulated by many microenvironmental regulatory mechanisms. Studies on advanced HBV-related liver fibrosis have found that both intrahepatic Th17 cells and Tregs are increased in progressive HBV-associated fibrosis. Additionally, activated HSCs can increase the number of Th17 cells and Tregs through the PGE2/EP2/EP4 pathway (18). Therefore, in the presence of HBV-induced liver fibrosis, HSCs have been found to be more than just end-stage effector cells of fibrosis; they can also actively alter the local immune microenvironment. Thus, Tregs are transformed into functional components of the fibrotic microenvironment and no longer function merely as regulators responding to inflammation; rather, in the context of advancing HBV-related liver fibrosis, a mutually reinforcing closed regulatory loop of “HSC activation – immune remodeling – fibrosis persistence” may arise, where Tregs serve as a key intermediary, being both induced by the microenvironment and actively contributing to its construction.

In short, the function of Tregs in HBV-associated liver fibrosis should be understood in light of the microenvironment of a dynamic Treg/Th17 imbalance and HSC activation, rather than being judged solely by changes in Treg numbers.

### 2.4 Treg functional reprogramming and immunosuppressive network formation in HBV-associated cirrhosis

HBV-related liver cirrhosis is the end-stage structural pathological condition of prolonged progression of chronic HBV infection. The diseases of the liver that occur are generally irreversible, such as cirrhosis, portal hypertension and declining liver function reserve. Cirrhosis is not an irreversible pathological change of the liver; instead, it is an immunopathological state that features immune tolerance, chronic inflammation, a pro-fibrotic microenvironment and intrahepatic immunosuppression. Therefore, Tregulatory cells have shown more pronounced changes in quantity, shape and function. Thus, the two will work together to reduce immune damage and increase immunosuppression simultaneously in advanced cirrhosis.

In light of the above modifications, Treg function in HBV-associated cirrhosis is no longer a static phenomenon but rather a stage-by-stage, non-linear renovation process. In the first stage, Tregs largely retain the immunoregulatory function they have acquired during chronic hepatitis and hepatic fibrosis by suppressing excessive activation of effector T cells and limiting immune-mediated injury to hepatocytes, but may also suppress HBV-specific antiviral responses. In the second stage, the cirrhotic intrahepatic microenvironment gradually induces a Treg phenotype and function. Persistent exposure to viral antigens promotes the activation of hepatic stellate cells, extracellular matrix deposition and an environment rich in TGF-β, IL-6, IL-10 and PGE2; these factors collectively impair Treg recruitment, stability, migration and suppressive function. Activated hepatic stellate cells can promote the differentiation of both Th17 cells and Tregs via the PGE2/EP2/EP4 pathway, providing a direct mechanistic basis for this microenvironment-induced immune dysregulation (18). In the third stage, Tregs may be incorporated into an intrahepatic immunosuppressive-fibrotic circuit and thus contribute to the maintenance of local immune tolerance, effector-cell dysfunction, and persistence of the fibrotic niche. This model does not assume a fixed linear sequence of all patients; instead, it provides a framework for interpreting heterogeneous Treg results as different stages of the disease, immune environment and functional status during HBV infection-cirrhosis.

In terms of quantitative changes, the levels of Tregs in the peripheral blood and liver of patients with HBV-associated cirrhosis are not entirely consistent, and this discrepancy itself has biological significance. Reports from cirrhosis cohorts have described Treg/Th17 imbalance as well as reduced or dysfunctional Tregs (19), and Treg enrichment has also been reported in HBV-associated HCC (20). HCC observation should not be directly combined with cirrhosis data because the tumour microenvironment has added immune selection pressure that is not present in cirrhosis alone. The above-mentioned seemingly different results can be analysed more systematically in three ways. The first axis is anatomical subdivision. Peripheral blood and liver tissue are different immune environments, and circulating Tregs do not fully reflect the status of intrahepatic Tregs. Peripheral Tregs are mainly involved in systemic immune regulation; intrahepatic Tregs are directly exposed to persistent HBV antigens, activated hepatic stellate cells, extracellular matrix remodelling, sinusoidal architectural distortion, and myeloid-cell-derived regulatory signals (7, 21, 22). Therefore, reduced or functionally impaired circulating Tregs do not necessarily exclude local Treg enrichment or enhanced suppressive activity within the cirrhotic liver. The second axis is disease trajectory. A recent single-cell transcriptomic study that included HBV infection, cirrhosis, and HBV-associated HCC reported that Treg proportions decreased from HBV infection to cirrhosis but increased after transition to HCC, together with expansion of exhausted CD8^+^ T cells (23). In the present cirrhosis-focused review, the HCC arm is used only as adjacent evidence showing that late tumor-associated immune remodeling can reverse the direction of Treg change; it is not treated as part of the HBV infection–cirrhosis continuum. This observation helps explain why cross-sectional studies that combine different advanced-disease states may report opposing Treg patterns, while preserving a clear distinction between cirrhosis and HCC. The third axis is methodological and phenotypic heterogeneity: different studies have defined Tregs using markers ranging from broad CD4^+^ CD25^+^ criteria to more specific subsets such as memory Tregs or CTLA-4^+^ FOXP3^+^ Tregs, and these populations are not functionally equivalent, as detailed below in the discussion of phenotypic heterogeneity. Thus, Treg changes in HBV-associated cirrhosis should be interpreted by integrating anatomical compartment, disease trajectory up to cirrhosis, phenotypic subset, functional integrity, and antiviral treatment background, rather than by comparing Treg frequency alone.

From the perspective of phenotypic heterogeneity, Tregs in HBV-associated cirrhosis are not a homogeneous population but instead display pronounced heterogeneity. In addition to classical CD4^+^ CD25^+^ FOXP3^+^ Tregs, multiple Treg subsets—including memory Tregs (mTregs; CD45RA⁻FOXP3^hi), CTLA-4-high Tregs, C-X-C chemokine receptor type 5 (CXCR5)-associated follicular Tregs (Tfr), and Treg subpopulations with migratory and tissue-resident features—have been reported to be associated with chronic HBV infection and cirrhosis progression. Some studies have shown that mTregs are selectively increased during the inflammatory active phase and are associated with ALT, bilirubin levels, and inflammatory status (13). An increase in CTLA-4^+^ FOXP3^+^ Tregs may indicate a more pronounced immunosuppressive state and has been associated with HBV DNA levels, antiviral treatment responses, and poor prognosis (24). Preliminary studies suggest that decreased expression of transcriptional regulatory factors such as special AT-rich sequence-binding protein 1 (SATB1) in Tregs may affect their stability and their capacity to regulate antiviral immune responses (25). These findings suggest that Treg alterations during cirrhosis should not be simply summarized as an “increase” or “decrease”, but should instead be evaluated in terms of subset composition, activation status, migratory capacity, and suppressive function.

Disruption of the Treg/Th17 balance is one of the main immunological abnormalities in HBV-associated cirrhosis. Several studies have shown that patients with HBV-associated cirrhosis often have increased Th17 cells and a reduced Treg/Th17 ratio. This ratio is closely associated with the degree of liver damage, markers of inflammation, the extent of fibrosis and clinical outcome (14, 18, 19, 26–28). Th17 cells promote hepatic inflammation and HSC activation by releasing pro-inflammatory factors such as IL-17, IL-21 and IL-23, and Tregs suppress immune activation through IL-10, TGF-β, CTLA-4 and other immunosuppressive pathways (28, 29). Sustained antigen exposure in cirrhosis can cause the production of inflammatory factors and damage to the structure of the liver, altering the balance of regulatory T cells (Tregs) and T helper 17 cells (Th17), and turning the Treg/Th17 axis into a driver of chronic inflammation and disease development rather than a suppressor of the immune system. Treg/Th17 imbalance is associated with the severity and short-term prognosis of HBV-related acute-on-chronic liver failure and decompensated cirrhosis, and thus this axis may serve as an important biological window for evaluating the immune status of cirrhosis and predicting clinical outcomes (26, 30).

At the level of function, Tregs in HBV-associated cirrhosis have a mixed profile that includes both enhanced immunosuppressive activity and intrinsic cellular dysfunction. On the one hand, intrahepatic Tregs can inhibit the function of CD8^+^ effector T cells and NK cells by releasing IL-10 and TGF-β, and by highly expressing inhibitory molecules such as CTLA-4 and PD-1; thus, the antiviral immune response is weakened, and conditions that favour chronic HBV infection and immune evasion are created. Some research has shown that Tregs may impair NK-cell cytotoxicity by inducing the expression of inhibitory receptors on NK cells and thus form a Treg-mediated innate immunosuppressive axis (21–23). At the same time, extended inflammatory stress, oxidative damage and metabolic disorders in cirrhosis can reduce the stability and survival of Tregs by inducing apoptosis, thus impairing their suppressive function (31). Therefore, the “functional remodelling” of Tregs in cirrhosis should not be interpreted as an increase in immunosuppressive power; rather, it is an all-too-comprehensive reflection of multiple defects at the level of Treg quantity, shape, metabolism and suppression capacity. The many deficiencies may be due to the combined effect of continuous antigen exposure, inflammatory stress and altered metabolism in a cirrhotic liver. Prolonged exposure to HBV antigens and damage-associated inflammatory signals may continuously stimulate Treg cells, thus inhibiting T-cell function and impairing effector T cells. Oxidative stress, mitochondrial damage and altered redox regulation will also shorten the life and impair the function of Tregs. Dysregulation of the nuclear factor erythroid 2–related factor 2/heme oxygenase-1 (Nrf2/HO-1) axis has been linked to impaired antioxidant defence and reduced Treg fitness in liver disease (31). Deposition of extracellular matrix and remodelling of sinuses can also alter chemokine gradients and tissue-retention signals to promote the accumulation of specific Treg subsets in fibrotic tissue. Under the above circumstances, Tregs may show an increased expression of inhibitory molecules and simultaneously have impaired survival, altered transcriptional stability, or reduced regulatory precision. This can help explain why Tregs in HBV-associated cirrhosis appear immunosuppressive at the network level but are dysfunctional at the cellular level.

**Figure 2:**
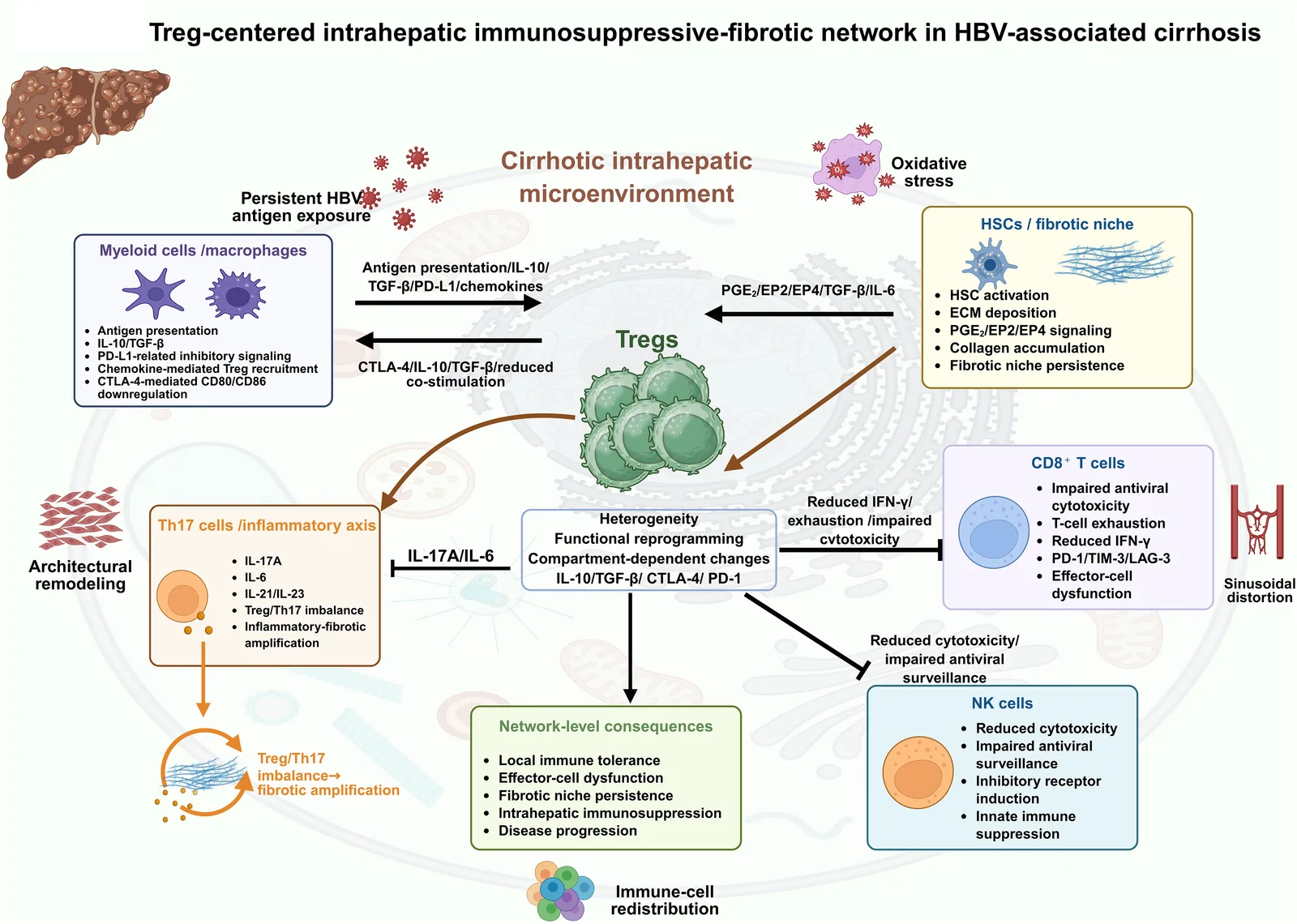
Treg-centered intrahepatic immunosuppressive-fibrotic network in HBV-associated cirrhosis. The schematic depicts interactions of regulatory T cells (Tregs) with myeloid cells/macrophages, hepatic stellate cells (HSCs), T helper 17 (Th17) cells, CD8^+^ T cells, and natural killer (NK) cells within the cirrhotic liver. Persistent HBV antigen exposure, oxidative stress, extracellular matrix remodeling, sinusoidal distortion, and immune-cell redistribution shape Treg recruitment and function. The resulting Treg-centered network contributes to local immune tolerance, impaired antiviral surveillance, effector-cell dysfunction, Treg/Th17 imbalance, and persistence of the fibrotic niche. Arrows indicate stimulatory or regulatory interactions, whereas blunt-ended lines indicate inhibitory effects. EP2, prostaglandin E receptor 2; EP4, prostaglandin E receptor 4; LAG-3, lymphocyte activation gene-3; PD-1, programmed cell death protein 1; TIM-3, T-cell immunoglobulin and mucin-domain containing-3. Created with BioRender.com.

The microenvironment of disease in the liver serves as an external regulator for Treg cells. In the context of HBV-related cirrhosis, activated hepatic stellate cells, inflammation and changes in sinusoidal structure lead to an immunosuppressive microenvironment; HSCs can promote the expansion of both Tregs and Th17 cells through the PGE2/EP2/EP4 pathway, and they work together with other mediators such as TGF-β, IL-6 and IL-17 to drive fibrosis and inflammation (18). Single-cell studies have provided specific support for the idea that the cirrhotic immune niche is characterised by myeloid-lymphoid crosstalk. Scar-associated macrophage subsets, such as macrophage-CD9/IL18 and macrophage-C1QA populations, are selectively expanded in HBV-associated cirrhotic liver tissue, and computational cell-cell communication analysis predicts enhanced signalling among myeloid cells and multiple immune compartments (22). These changes occur simultaneously with impaired NK-cell cytotoxicity, and thus cirrhosis is not solely a problem of Treg alteration but rather a coordinated remodelling of myeloid cells, Tregs, NK cells and effector T cells (21, 22). Mechanistically, myeloid cells may promote Treg recruitment and functional maintenance by antigen presentation, IL-10, TGF-β, programmes death-ligand 1 (PD-L1)-related inhibitory signals, and chemokine-mediated trafficking; Tregs can reciprocally suppress the maturation and co-stimulatory capacity of antigen-presenting cells (APCs) through CTLA-4-dependent downregulation of CD80/CD86, as well as IL-10- and TGF-β-mediated inhibition. Antigenic stimulation of HBV and signaling by HBx proteins may also enhance the interaction between hepatic stellate cells and immune cells to exacerbate local immune dysregulation (32, 33). All of the above indicate that, rather than functioning independently, Tregs and the myeloid compartment constitute a combined regulatory node in the intrahepatic immunosuppressive and pro-fibrotic network of HBV-associated cirrhosis. This node is clinically relevant; thus, targeted modulation of intrahepatic Tregs or their myeloid-cell interactions could potentially affect multiple immune-fibrotic pathways simultaneously, but such strategies have yet to be verified in mechanism and clinical studies. In short, the above results support a model where functionally reprogrammed Tregs serve as a central regulatory node for myeloid-cell crosstalk, HSC-driven fibrotic niche formation, effector-cell dysfunction, NK-cell suppression and Treg/Th17 imbalance in HBV-associated cirrhosis (**Figure 2**).

## 3 Treg-targeted immunomodulatory strategies for HBV-associated liver disease

The role of Tregs in HBV infection, as well as in HBV-associated hepatic fibrosis and cirrhosis, is strongly stage dependent. While Tregs restrain excessive immune activation and mitigate immune-mediated hepatocyte injury, they may also attenuate HBV-specific effector T-cell immunity, thereby participating in viral persistence, immune tolerance, and preservation of the intrahepatic immunosuppressive milieu. Accordingly, immunoregulatory strategies targeting Tregs should not be reduced to a binary approach of “boosting” or “suppressing” Treg activity. Rather, they require stage-specific stratification that considers viral replication, inflammatory activity, fibrosis or cirrhosis progression, and concomitant antiviral treatment.

### 3.1 Treg depletion or suppression for the restoration of antiviral immune responses

#### 3.1.1 Anti-CD25 monoclonal antibodies

CD25, the α chain of the IL-2 receptor, is one of the commonly used surface markers of FOXP3^+^ Tregs and has also been a molecule of interest in early studies targeting Tregs.Anti-CD25 monoclonal antibodies, such as daclizumab, have been clinically investigated in immune-related diseases including multiple sclerosis. They block the binding of IL-2 to the high-affinity IL-2 receptor, thereby affecting T-cell activation, Treg expansion, and functional maintenance. Daclizumab was previously used for the prevention of transplant rejection and in non-HBV diseases such as multiple sclerosis; however, its effects are not Treg-specific, as CD25 is also expressed on activated effector T cells (34, 35). Therefore, anti-CD25 intervention has clear limitations in selectivity and is accompanied by concerns regarding immune imbalance and safety.

In HBV-related research, evidence supporting anti-CD25 strategies mainly derives from *in vitro* mechanistic studies. Stoop et al. found that depletion of CD25^+^ cells from PBMCs of patients with chronic HBV infection enhanced T-cell proliferative responses upon HBV antigen stimulation (12). Xu et al. also reported that removal of CD4^+^ CD25^+^ Tregs increased IFN-γ production by PBMCs following HBV antigen stimulation, suggesting that CD25^+^ Tregs participate in maintaining HBV-specific T-cell hyporesponsiveness (7). In addition, HBcAg-specific CD4^+^ CD25^+^ Tregs have been reported to contribute to immune tolerance and acute exacerbation during chronic HBV infection (11).

However, it should be emphasized that the current evidence does not support the direct use of anti-CD25 strategies as a therapeutic approach for HBV-related liver disease. The major limitations include the following: first, CD25 targeting lacks Treg specificity and may also affect antiviral effector T cells; second, systemic Treg depletion may exacerbate immune-mediated hepatitis; and third, no large-scale clinical studies in HBV have yet validated the efficacy or safety of anti-CD25 therapy. Therefore, anti-CD25 intervention is more appropriately regarded as an experimental tool for demonstrating the involvement of Tregs in HBV-related immunosuppression, rather than as a clinically recommended intervention strategy at the present stage.

#### 3.1.2 Immune checkpoint inhibitors

PD-1 and CTLA-4 are important negative immunoregulatory molecules in chronic viral infection. CTLA-4 is highly expressed on Tregs and can inhibit CD28-mediated co-stimulatory signaling by competitively binding to, or removing, CD80/CD86 from the surface of antigen-presenting cells, representing one of the key mechanisms by which Tregs exert contact-dependent immunosuppression (36). PD-1 is broadly involved in T-cell exhaustion, peripheral tolerance, and negative immune regulation during chronic infection (37). Theoretically, blockade of PD-1 or CTLA-4 may help restore partial HBV-specific T-cell function; however, in HBV-related liver disease, this strategy must be approached with particular caution because of the risk of immune-mediated liver injury.

Clinical evidence for PD-1/CTLA-4 blockade in HBV-infected populations derives largely from HCC treatment rather than from therapy of chronic HBV infection or HBV-associated cirrhosis. FDA-approved HCC regimens (38, 39) and safety data from HBV-infected patients receiving anticancer therapy (40, 41) are cited here only to define the clinical boundary of this approach: HBV screening, antiviral prophylaxis when indicated, and monitoring of HBV DNA and liver function are required when immune checkpoint inhibitors are used for oncological indications. These data do not establish checkpoint inhibitors as Treg-directed or antiviral therapy for patients with chronic HBV infection or compensated cirrhosis without HCC. In these non-tumor populations, checkpoint blockade remains insufficiently supported and should be considered only within rigorously monitored clinical research.

#### 3.1.3 Low-dose cyclophosphamide and CCR4-targeted strategies

Low-dose or metronomic cyclophosphamide has been explored in cancer immunology as a means to deplete Tregs and potentiate antitumor immunity. In patients with advanced malignancies, Ghiringhelli and colleagues reported that metronomic cyclophosphamide selectively decreased CD4^+^ CD25^+^ Tregs while restoring T-cell and NK-cell effector activity (42). Nevertheless, these findings come primarily from oncological immunotherapy settings, which are clearly distinct from HBV-associated liver disease. Given that patients with chronic HBV infection and cirrhosis may already exhibit immune dysregulation, impaired bone marrow reserve, and heightened susceptibility to infection, the safety of low-dose cyclophosphamide as a long-term immunoregulatory approach remains insufficiently established.

C-C chemokine receptor type 4 (CCR4) is another molecule of interest in Treg-targeted research. Effector Tregs and some tumor-infiltrating Tregs can highly express CCR4, and the anti-CCR4 monoclonal antibody mogamulizumab exerts its effects by depleting CCR4^+^ malignant T cells and CCR4^+^ Tregs. It has been used in diseases such as adult T-cell leukemia/lymphoma and cutaneous T-cell lymphoma (43, 44). However, there is currently almost no direct evidence that CCR4-targeted strategies can safely and effectively modulate intrahepatic Tregs in HBV-related liver disease. Given that CCR4 is not an HBV-specific Treg marker, systemic depletion of CCR4^+^ cells may cause broad immune perturbation.

### 3.2 Augmentation or stabilization of Treg activity for the containment of immune-mediated hepatic damage

#### 3.2.1 Low-dose IL-2 therapy

IL-2 is a key signaling molecule required for Treg survival, proliferation, and maintenance of FOXP3 expression. Because Tregs highly express CD25 and are relatively sensitive to low concentrations of IL-2, low-dose IL-2 is considered capable of relatively selective Treg expansion. Clinical studies of low-dose IL-2 have been conducted in diseases such as chronic graft-versus-host disease and systemic lupus erythematosus, showing its potential to expand Tregs and improve immune homeostasis (45, 46). In the field of autoimmune hepatitis, reviews have also proposed that low-dose IL-2 may ameliorate abnormal immune-mediated injury by enhancing Tregs, although the evidence remains exploratory (47).

Low-dose IL-2 is used to reduce excessive immune-mediated damage by expanding the number of regulatory T cells (Tregs) in HBV-associated liver disease, and it is theoretically suitable for conditions dominated by immune-mediated injury, such as acute liver injury, chronic inflammatory activity, or acute-on-chronic liver failure. Another characteristic of extended HBV infection is persistent viral replication and an impaired anti-viral immune response; additionally, Treg expansion induced by low-dose IL-2 can suppress HBV-specific effector T-cells and thus be harmful to viral control.

This conflict can be shown as a time delay. Low-dose IL-2 is more likely to provide a net benefit in the presence of acute immune-mediated liver injury, and restraining excessive inflammation is the primary therapeutic goal here. Transient enhancement of Treg activity may thus reduce hepatocyte damage to some extent and is particularly relevant in certain patients with HBV-related acute-on-chronic liver failure who have exhibited Treg/Th17 imbalance and a poorer short-term prognosis (30). HBV infection enters a chronic antigen-persistent state that shows antiviral hyporesponsiveness, and further expansion of Tregs may strengthen immune tolerance and inhibit the recovery of HBV-specific effector T cells. Thus, the same intervention may have different effects according to the time of the disease, antigen load, inflammatory status and liver function.

Therefore, low-dose IL-2 should be used in the context of HBV-associated liver disease as a stage-specific immunomodulatory strategy requiring further verification, rather than as a general Treg-enhancing therapy. Future research will specify the target population, timing of administration and dosing window, background antiviral therapy precisely, and simultaneously monitor HBV DNA, quantitative HBsAg, ALT, bilirubin, INR, infection markers, Treg/Th17 balance, and HBV-specific T-cell function. At present, low-dose IL-2 is not recommended for chronic HBV infection, HBV-related fibrosis and HBV-associated cirrhosis as routine treatment.

#### 3.2.2 mTOR inhibitors

The mechanistic target of rapamycin (mTOR) signalling pathway is involved in T-cell differentiation, metabolic reprogramming and the functional stability of Tregs. Under short-term exposure or in certain circumstances, mTOR inhibition can increase FOXP3 expression and generate a Treg-like phenotype to suppress the proliferation of effector T cells to some extent (48). Rapamycin/Sirolimus are commonly employed in transplant immunology and have also been shown to suppress the expansion and function of Tregs (49).

Regulation of Tregs by the mTOR pathway cannot be reduced to “mTOR inhibition = Treg expansion”. Different mTOR complexes, varying degrees of inhibition, different durations of exposure and other factors in the inflammatory microenvironment can all affect the stability, migration and effector functions of Tregs. Therefore, in HBV-related liver disease, it is not reasonable to refer to rapamycin as a treatment for “stabilising Treg function”. A more appropriate statement is that the mTOR-Treg axis may be a possible mechanism of immunometabolic remodelling in chronic HBV infection and cirrhosis, but direct human evidence is lacking.

Rapamycin is a general immunosuppressant, and thus may be more susceptible to infection. Given that the patient has HBV infection, immunosuppressive drugs should be avoided unless there is an urgent need and the risk of reactivation is low. Therefore, mTOR inhibitors should not be used clinically to modulate Tregs in HBV-related liver disease at present, but rather serve as a direction for future mechanistic studies.

#### 3.2.3 Vitamin D- and retinoic acid-related strategies

Vitamin D/vitamin D receptor (VDR) signaling and all-trans retinoic acid (ATRA) are both associated with the differentiation balance between Tregs and Th17 cells. ATRA can promote TGF-β-induced differentiation of FOXP3^+^ Tregs and inhibit Th17-cell formation (50, 51). Vitamin D/VDR signaling is also considered to participate in immune regulation, and some studies have shown that hepatic VDR expression is reduced in patients with chronic HBV infection and is associated with the degree of liver inflammation and fibrosis (52).

At present, certain correlations have been observed among serum vitamin D levels, hepatic VDR expression, and inflammation/fibrosis in patients with chronic HBV infection. However, these associations do not yet prove that vitamin D supplementation can improve the progression of HBV-related liver disease through Treg regulation. Evidence that ATRA modulates Treg/Th17 differentiation is derived mainly from animal and *in vitro* immunological studies, and HBV-related clinical validation is also lacking.

### 3.3 Balance-regulating strategies targeting the Treg/Th17 axis

Dysregulation of the Treg/Th17 axis represents a relatively prominent immunological hallmark of HBV-associated hepatic fibrosis and cirrhosis. Rather than merely augmenting or suppressing Tregs, recalibrating the Treg/Th17 axis more accurately embodies an immune homeostasis-oriented therapeutic strategy. Nevertheless, direct clinical evidence supporting Treg/Th17-targeted interventions for HBV-associated hepatic fibrosis or cirrhosis is still limited.

#### 3.3.1 Blockade of the IL-17 pathway

IL-17A is a typical effector cytokine that promotes inflammation and fibrosis in Th17 cells. Li and others found that both peripheral Tregs and Th17 cells were increased in patients with HBV-associated liver fibrosis, and a lower Treg/Th17 ratio was associated with more severe liver injury and progression of fibrosis. *In vitro* experiments show that IL-17 promotes the proliferation and activation of HSCs, and CD4^+^ CD25^+^ cells inhibit HSC activation (14). Other studies have also shown that IL-17 promotes the progression of liver fibrosis by affecting Kupffer cells and HSCs (53, 54).

Blockade of the IL-17 pathway will thus reduce inflammation-induced activation of HSCs and ECM deposition. IL-17A blockade or targeting of the IL-17 pathway in animal experiments has been used to reduce inflammation and fibrosis in models of cholestasis and chemical-induced liver injury (55). The above evidence is only based on animal studies. The current anti-IL-17A and anti-IL-23 drugs are mainly used to treat immune-mediated inflammatory diseases such as psoriasis, ankylosing spondylitis and psoriatic arthritis, but there is no supporting evidence for their application in the treatment of HBV-related liver fibrosis or cirrhosis at present.

Most clinical experience with IL-17 pathway inhibition has been accumulated in non-hepatic immune-mediated inflammatory diseases. Secukinumab and ixekizumab are representative anti-IL-17A monoclonal antibodies used to treat psoriasis, psoriatic arthritis and spondyloarthritis in recent years. In general, these agents are not associated with broad cytotoxic immunosuppression; however, a consistent safety signal has emerged: an increased risk of mucocutaneous Candida infection due to the physiological role of IL-17 in antifungal mucosal defence (56, 57). HBV-associated cirrhosis is more likely to develop such complications because immune dysfunction in cirrhosis includes both systemic inflammation and immunodeficiency; therefore, it is highly susceptible to infectious complications (58). The risk of HBV reactivation in HBV-infected patients taking biologic immunomodulators should not be ignored; based on real-world clinical data for secukinumab in patients with psoriasis and HBV/HCV infections, a pre-treatment baseline viral load measurement and prophylactic antiviral therapy may be necessary (59).

A relatively weaker inhibition of the IL-17 pathway may be needed. Secukinumab and ixekizumab selectively target IL-17A, and safety data for these agents indicate that infection-related events should be monitored, especially mucocutaneous Candida infections (56, 57). Bimekizumab is a dual inhibitor of IL-17A and IL-17F; in a head-to-head Phase 3 trial for plaque psoriasis, it outperformed secukinumab clinically but had a higher incidence of oral candidiasis due to the broader suppression of the IL-17A/F axis (60). Brodalumab further downregulates IL-17RA by blocking it and thus inhibits the signal transduction of multiple IL-17 family cytokines, including IL-17A, IL-17F and IL-17E (61). The above mechanisms suggest that IL-17A-selective blockade, IL-17A/F dual blockade and IL-17RA blockade should not be considered interchangeable in terms of their potential translation to HBV-associated liver disease.

Therefore, blockade of the IL-17 pathway may be considered a mechanistically feasible but clinically unvalidated anti-fibrotic immunological strategy for HBV-related liver fibrosis. It may suppress Th17-mediated inflammation and HSC activation to reduce inflammatory-fibrotic amplification. Several problems need to be solved before clinical application: Does IL-17 blockade inhibit antiviral, antifungal or mucosal host defence in HBV-infected patients? Is there an increased risk of infection due to cirrhosis-associated immune dysfunction? Is dual blockade of IL-17A/F or IL-17RA blockade superior to selective IL-17A inhibition? And can suppressing Th17 responses restore the Treg/Th17 balance instead of causing a new type of immune deviation? For the reasons above, IL-17 pathway blockade should be limited to experimental research at present and is not recommended for HBV-related fibrosis or cirrhosis in non-clinical settings.

#### 3.3.2 RORγt inhibition

Retinoic acid receptor-related orphan receptor γt (RORγt) is the master transcription factor governing Th17-cell differentiation and can induce the expression of Th17-related effector molecules such as IL-17A and IL-17F (62). Therefore, RORγt inhibitors may theoretically improve Treg/Th17 imbalance indirectly by suppressing Th17 differentiation at an upstream level. Compared with direct IL-17A blockade, RORγt inhibition is more oriented toward regulating the Th17 differentiation program rather than targeting a single cytokine.

In recent years, RORγt inverse agonists have shown potential in chronic carbon tetrachloride (CCl_4_)-induced liver injury models to reduce intrahepatic immune cell infiltration and attenuate liver injury and fibrosis (63). However, this evidence remains limited to animal experiments and is not derived from HBV-related models. At present, there are no clinical studies of RORγt inhibitors in patients with HBV-related liver fibrosis, HBV-associated cirrhosis, or chronic HBV infection. Therefore, in this review, RORγt inhibition should be positioned as an experimental strategy for upstream regulation of the Th17 axis and should not be emphasized as a near-term direction for clinical translation.

### 3.4 Combined immunomodulation in the context of antiviral therapy

The foundation of treatment for chronic HBV infection remains effective suppression of viral replication. Current first-line antiviral therapies include nucleos(t)ide analogues (NAs), such as entecavir and tenofovir, as well as pegylated interferon-α (PEG-IFN-α) for selected patients. If Treg-related immunomodulation is to enter clinical investigation, it must be built upon adequate antiviral therapy and virological monitoring.

#### 3.4.1 Effects of nucleos(t)ide analogues on Tregs

NAs can effectively suppress HBV replication, but their capacity for direct immune reconstitution is limited. In a longitudinal study of HBeAg-positive patients with chronic hepatitis B, Zhang et al. found that entecavir-induced suppression of HBV replication was accompanied by increased Th17 cells, decreased Tregs, and a reduced Treg/Th17 ratio (64). Tian et al. also observed that entecavir treatment affected CD4^+^ T-cell subsets, including changes in Th17 cells and Tregs (65). These findings suggest that a decline in viral load may be accompanied by partial remodeling of the Treg/Th17 axis.

However, findings across studies are not entirely consistent. Some studies have shown that although entecavir treatment can reduce HBV DNA levels and improve liver function, it does not significantly alter the proportions of certain immune cell populations or cytokine levels (66). In addition, NA therapy generally fails to fully restore HBV-specific T-cell function, suggesting that viral suppression alone is insufficient to completely reverse the immune hyporesponsive state in chronic HBV infection.

#### 3.4.2 Effects of interferon-α on Tregs and its potential for combination therapy

PEG-IFN-α has both antiviral and immunomodulatory effects and can enhance innate and adaptive immune responses. Some studies suggest that HBsAg clearance is associated with changes in Treg/Th17 status or HBV-specific T-cell function, although most of this evidence comes from small-sample or observational studies (67). Other studies have shown that PEG-IFNα-2b can enhance HBV-specific CD8^+^ T-cell responses and modulate the T-cell immune status in patients with chronic hepatitis B (68).

However, there is currently insufficient evidence to support PEG-IFN-α combined with Treg-targeted intervention as a therapeutic strategy for HBV-related liver disease. PEG-IFN-α itself may cause adverse events such as hepatitis flare, fever, bone marrow suppression, and autoimmune reactions; if further combined with Treg inhibition or immune checkpoint blockade, the risk of liver injury may increase. Although PEG-IFN-α may be associated with Treg status, future studies may explore immune stratification and combination strategy design on the basis of antiviral therapy, but such approaches cannot yet be recommended as routine practice.

### 3.5 Current limitations and future directions

#### 3.5.1 Limitations of the current evidence base

Several limitations of the current evidence base should be acknowledged. First, much of the available human evidence is cross-sectional or observational. Associations between peripheral or intrahepatic Treg measures and virological, inflammatory, or fibrosis-related parameters therefore do not establish whether Treg remodeling drives disease progression, results from the changing hepatic microenvironment, or both. Interpretation is further limited by heterogeneity in disease-stage definitions, antiviral-treatment status, sample compartment, Treg identification strategies, and clinical endpoints. Second, HBV-specific interventional evidence remains scarce. None of the studies summarized in **Table 1** directly tested a Treg-targeted intervention in HBV-related fibrosis or HBV-associated cirrhosis; much of the translational rationale is extrapolated from non-HBV immune-mediated diseases, transplantation, preclinical models, or non-Treg-directed HBV studies. These limitations preclude firm conclusions regarding clinical efficacy, the optimal disease stage for intervention, and safety. Longitudinal studies with paired blood and liver sampling, standardized immune phenotyping, and stage-stratified HBV cohorts are needed, followed by mechanistically informed HBV-specific trials that incorporate virological control, fibrosis-related outcomes, hepatitis flares, infection, and hepatic decompensation as prespecified endpoints.

**Table 1:** Representative clinical and translational evidence informing Treg- and Treg/Th17-related immunomodulatory strategies relevant to HBV-associated liver disease.

Strategy	Representative evidence	Main findings	Relevance to HBV-associated liver disease	References
Low-dose IL-2	Clinical study in chronic graft-versus-host disease	Low-dose IL-2 restored Treg homeostasis and supported Treg expansion in an immune injury-dominant setting	Provides clinical precedent for pharmacological Treg augmentation, but does not establish efficacy or safety in HBV infection or cirrhosis	(45)
Low-dose IL-2	Open clinical trial across autoimmune diseases	Low-dose IL-2 showed immunological and clinical effects across multiple autoimmune diseases, supporting its role as a Treg-enhancing approach	Supports the concept of regulatory immune restoration, but the risk-benefit profile may differ in chronic antigen-persistent HBV infection	(46)
Low-dose IL-2 after liver transplantation	Clinical trial in liver transplant recipients during immunosuppression withdrawal	Low-dose IL-2 expanded circulating Tregs but failed to expand donor-specific Tregs or promote liver allograft tolerance, and was associated with rejection events	Demonstrates that systemic expansion of circulating Tregs alone may be insufficient for liver-localized immune tolerance	(69)
Treg-selective IL-2 pathway agonist	Phase 1 studies of rezpegaldesleukin/NKTR-358 in healthy volunteers and patients with systemic lupus erythematosus	NKTR-358 induced sustained and selective Treg expansion	Suggests that engineered IL-2 pathway agonists may improve Treg selectivity, but no HBV- or cirrhosis-specific data are available	(70)
IL-17A blockade: secukinumab	Safety review of secukinumab and real-world study in psoriasis patients with HBV or HCV infection	Secukinumab has accumulated non-hepatic clinical safety data; mucocutaneous Candida infection and HBV reactivation monitoring are relevant safety considerations	Provides indirect safety information for IL-17A blockade; baseline HBV screening, antiviral prophylaxis when indicated, and HBV DNA/liver function monitoring are important in HBV-infected patients	(56, 59)
IL-17A blockade: ixekizumab	Integrated long-term safety analysis from randomized clinical trials in psoriasis, psoriatic arthritis, and axial spondyloarthritis	Long-term data support infection monitoring; mucocutaneous Candida infection remains a pathway-relevant safety signal	Supports class-level caution for IL-17A blockade, but does not address HBV-associated fibrosis, cirrhosis, or impaired hepatic reserve	(57)
IL-17A/F dual blockade: bimekizumab	Head-to-head phase 3 trial comparing bimekizumab with secukinumab in plaque psoriasis	Bimekizumab showed greater clinical efficacy than secukinumab but was associated with more oral candidiasis	Highlights that broader IL-17A/F blockade may increase mucosal antifungal risk, which is relevant when considering patients with cirrhosis-associated immune dysfunction	(60)
IL-17RA blockade: brodalumab	Evidence-based review of brodalumab in moderate-to-severe psoriasis	Brodalumab blocks IL-17RA and thereby inhibits signaling mediated by multiple IL-17 family cytokines	Demonstrates that receptor-level IL-17 blockade is mechanistically broader than IL-17A-selective inhibition; translation to HBV liver disease would require separate safety evaluation	(61)
CAR-Tregs or TCR-engineered Tregs	Translational evidence and reviews in liver disease, autoimmune liver disease, and transplantation tolerance	Engineered regulatory cell strategies may confer antigen or tissue specificity, but remain largely conceptual in HBV-associated liver disease	Provides a conceptual model for antigen- or tissue-directed Treg modulation; current evidence is not sufficient to support clinical use in HBV-associated cirrhosis	(71–73)

CAR-Treg, chimeric antigen receptor regulatory T cell; GVHD, graft-versus-host disease; HBV, hepatitis B virus; IL-2, interleukin-2; IL-17RA, interleukin-17 receptor A; SLE, systemic lupus erythematosus; TCR, T-cell receptor; Treg, regulatory T cell.

#### 3.5.2 Translational limitations of Treg-targeted intervention

Tregs are required regulatory cells of the immune system in HBV-associated liver disease, but directly targeting them has not yet been achieved in practice.

First, the consequences of Treg modulation are strongly dependent on disease stage. Over-suppression of Tregs in acute HBV infection or acute liver injury may enhance immune-mediated damage to the liver. Tregs in the immune-tolerant or hyporesponsive state of chronic HBV infection may promote viral persistence by indiscriminate expansion. Liver fibrosis and cirrhosis are characterized by the coexistence of chronic inflammation, immunosuppression, impaired anti-infective immunity, and defective tissue repair. Therefore, Treg-targeted intervention should be based on a full-process evaluation of the disease stage, viral replication, inflammatory status and hepatic functional reserve.

Second, peripheral Tregs cannot fully represent intrahepatic Tregs. The main pathological environment of HBV-associated cirrhosis is in the liver, and peripheral Tregs may differ from intrahepatic Tregs in terms of quantity, shape and activity. In the future, liver tissue analysis, single-cell sequencing, spatial transcriptomics, flow cytometry and functional experiments will be employed to define the characteristic features, tissue-resident status and interactions of intrahepatic Tregs with HSCs, myeloid cells, NK cells and effector T cells.

Third, there is a lack of tools for targeting HBV antigen-specific Tregs. Existing interventions mostly target broadly expressed molecules such as CD25, CTLA-4, CCR4, IL-2, or mTOR, making it difficult to distinguish HBV antigen-specific Tregs from Tregs that maintain normal immune homeostasis.

Fourth, safety remains a major bottleneck for clinical translation. In the context of HBV infection, enhancing effector immunity or weakening Tregs may trigger exacerbation of HBV-related immune-mediated hepatitis. Conversely, enhancing Tregs or using immunosuppressive agents may lead to inadequate viral control or an increased risk of infection.

Fifth, sex-related heterogeneity remains insufficiently characterized. Although sex differences are well recognized in HBV-related disease outcomes, including the risk of HCC, it remains unclear whether Treg abundance, phenotype, intrahepatic localization, or suppressive function are remodeled differently in male and female patients across the HBV infection-to-cirrhosis course. In murine HBV models, males exhibited weaker HBV-specific T-cell responses associated with higher intrahepatic Treg numbers (78), suggesting that Tregs may contribute to sex-related differences in antiviral immunity. However, this experimental observation has not been adequately validated in sex-stratified human cohorts, and sex-specific analyses of Treg remodeling remain limited in the available clinical literature. Future studies should prespecify sex as a biological variable, report sex-disaggregated immune data, and, where feasible, examine sex-by-disease-stage interactions while accounting for age, hormonal status, antiviral treatment, viral burden, and fibrosis stage.

Representative clinical and translational evidence informing Treg- and Treg/Th17-related immunomodulatory strategies is summarized in **Table 1**. Importantly, none of these studies directly evaluated Treg-targeted intervention in HBV-related fibrosis or HBV-associated cirrhosis; therefore, their relevance to HBV-associated liver disease remains indirect and hypothesis-generating.

### 3.6 Future clinical development pathways for Treg-targeted immunomodulation

Based on the above analysis, the current Treg-related strategies have had limited application to HBV-associated liver disease because most of them are systemic in nature and fail to address the issues of disease stage, viral antigen load and the balance between protective and virus-permissive immune regulation. In HBV-associated cirrhosis, the role of Tregs may be anti-inflammatory or immunosuppressive depending on the immune environment and actual circumstances; therefore, this defect is more pronounced here. Therefore, the future development of therapy will be personalised immunosuppression rather than broad-spectrum Treg modification. The following section will introduce four interconnected directions for this transition: biomarker-driven patient stratification, rational combination with antiviral therapy, antigen-specific and liver-targeted Treg modulation, and application in the context of liver transplantation (**Table 2**).

**Table 2:** Proposed clinical translation roadmap for Treg-targeted strategies in HBV-associated liver disease.

Development pathway	Strategy or stratification criterion	Target population or rationale	Translational status	Key references
Biomarker-driven trial design	Treg/Th17 ratio	May help distinguish inflammation-dominant states from relatively regulatory or suppressive immune states; useful for selecting between regulatory restoration and cautious attenuation of excessive Treg-mediated suppression	Conceptual trial-enrichment tool; associated with disease severity and prognosis in HBV-associated cirrhosis, but not yet validated as an interventional stratification marker	(14, 18, 19, 26–28, 30)
	Intrahepatic or peripheral Treg subset composition, including CTLA-4^+^FOXP3^+^ Tregs, and memory Tregs	May identify patients with enriched suppressive Treg phenotypes or altered Treg stability; supports more refined immune phenotyping beyond total Treg frequency	Descriptive and correlative evidence; not yet used prospectively for patient enrichment in HBV trials	(13, 24)
	HBV-specific effector T-cell function, IFN-γ production, and exhaustion markers	Helps determine whether impaired antiviral immunity, rather than uncontrolled necroinflammation, is the dominant immunological problem	Research assays are available, but routine clinical use and trial thresholds remain undefined	(7, 12, 37, 68)
Combination with novel antivirals	Sequential strategy: viral and antigen suppression first, followed by Treg-directed immunomodulation	Reduces antigen-driven immune exhaustion and Treg induction before attempting immune restoration; may provide a safer immunological window	Antigen-lowering antiviral combinations have entered clinical testing; sequencing with Treg-targeted agents remains untested	(74)
	Concurrent strategy: antiviral therapy and immunomodulation from the outset	May theoretically reduce antigenic stimulation and immune suppression simultaneously, but carries greater risk of immune-mediated liver injury	Should be restricted to carefully monitored early-phase trials; Treg-specific concurrent regimens have not been established	
Antigen-specific and liver-targeted Treg modulation	HBV antigen-specific Treg identification and monitoring	Addresses the lack of specificity in systemic Treg modulation; may identify Treg clones that selectively suppress HBV-specific antiviral responses	HBV core antigen-specific regulatory T cells have been reported, but therapeutic targeting remains technically challenging	(11, 75)
	CAR-Tregs or TCR-engineered Tregs	May confer antigen or tissue specificity on adoptively transferred regulatory cells; potentially relevant to autoimmune liver disease and transplantation tolerance	Conceptual in HBV-associated liver disease; more advanced experience comes from transplantation or non-HBV settings	(71–73)
	Nanoparticle-mediated intrahepatic delivery	May restrict immunomodulatory agents to hepatic immune or stromal compartments and reduce systemic immune perturbation	Liver-targeted nanomedicine has been explored in inflammation and fibrosis, but Treg-directed delivery has not been validated in HBV-associated cirrhosis	(76)
	FOXP3 stabilization through epigenetic or post-translational modulation	May restore Treg lineage stability when Treg dysfunction rather than Treg excess predominates	Supported mainly by transplantation models; requires caution in HBV because excessive Treg stabilization may favor viral persistence	(77)
	Treg-selective IL-2 pathway agonists or IL-2 muteins, such as rezpegaldesleukin/NKTR-358	May improve upon conventional low-dose IL-2 by preferentially expanding Tregs with less activation of non-regulatory T cells	Phase 1 evidence in healthy volunteers and systemic lupus erythematosus; not yet tested in HBV-associated liver disease	(70)
Liver transplantation context	Treg-mediated tolerance induction after liver transplantation	Aims to reduce long-term immunosuppression while preserving graft tolerance	Clinically challenging; low-dose IL-2 expanded circulating Tregs but failed to promote liver allograft tolerance and was associated with rejection events	(69)

CAR-Treg, chimeric antigen receptor regulatory T cell; FOXP3, forkhead box P3; HBV, hepatitis B virus; IFN-γ, interferon-γ; TCR, T-cell receptor; Treg, regulatory T cell.

#### 3.6.1 Biomarker-guided clinical trial design

A typical problem in the clinical application of Treg-targeting therapy is that patients have not been properly selected, despite the two-way role of Tregs in HBV-related liver disease. Rational Trial Design should distinguish between immune injury-dominant states and immune tolerance- or exhaustion-dominant states. Treg-enhancing strategies may be more suitable for clinical cases of extensive immune-mediated liver injury, such as acute liver injury, some cases of HBV-related acute-on-chronic liver failure, or decompensated cirrhosis with severe inflammatory activation, provided that the risk of infection and hepatic functional reserve are carefully considered. Cautious rebalancing of excessive Treg-mediated suppression may only be considered for patients who have achieved effective suppression of HBV replication but continue to exhibit persistent antigenemia, impaired HBV-specific CD8^+^T-cell function, enrichment of highly suppressive Treg subsets, and relatively preserved hepatic functional reserve. Therefore, the aim is not to decrease Tregs in general but to reduce the inhibitory function of Tregs on antiviral immune recovery selectively.

Future clinical trials will therefore add immune-related, virological and hepatic functional biomarkers to the stratification, enrichment and monitoring systems. These biomarkers should be selected based on the biological questions they are to address, and a single undifferentiated panel should not be employed. First, markers of immune injury and Treg/Th17 imbalance, such as peripheral and, when feasible, intrahepatic Treg/Th17 ratio, Th17-cell frequency, IL-17A, IL-6, ALT, bilirubin, and international normalized ratio (INR), can be used to identify patients with inflammatory liver injury. Second, markers of Treg-associated suppression, such as CTLA-4^+^ FOXP3^+^ Tregs, memory Treg subsets, IL-10, and TGF-β, can help to identify tolerance-dominant or immunosuppressive states (13, 24). Third, markers of antiviral effector competence, such as HBV-specific CD8^+^ T-cell function, IFN-γ production, cytotoxic markers and T-cell exhaustion markers, may indicate whether antiviral immune restoration is biologically feasible. The above exhaustion markers include PD-1, T-cell immunoglobulin and mucin-domain containing-3 (TIM-3), lymphocyte activation gene-3 (LAG-3), and T-cell immunoreceptor with immunoglobulin and immunoreceptor tyrosine-based inhibitory motif domains (TIGIT). Fourth, virological and antigen-burden markers such as HBV DNA, quantitative HBsAg, hepatitis B core-related antigen (HBcrAg), and serum HBV RNA (when available) should be employed to determine the antiviral background in which immunomodulation is performed. Finally, hepatic reserve and safety indicators should also be added to reduce the risk of hepatitis flares or liver decompensation; these include albumin, platelet count, Child-Pugh class, Model for End-stage Liver Disease (MELD) score, liver stiffness measurement (LSM), and non-invasive fibrosis indices such as the aspartate aminotransferase-to-platelet ratio index (APRI) or fibrosis-4 index (FIB-4). These markers should not be used solely as descriptive endpoints but rather as indicators of whether a patient has been damaged by immune-mediated injury, antiviral immune exhaustion, intrahepatic immunosuppression, or reduced hepatic reserve. In this way, we will not consider chronic HBV infection and HBV-associated cirrhosis as the same biological entity, and the opposite immune effects will not cancel each other out in an unselected group.

#### 3.6.2 Combination strategies with novel antivirals

Treg-directed immunomodulation is unlikely to be effective if implemented independently of antiviral therapy. Sustained exposure to HBV antigens promotes immune tolerance, T-cell exhaustion and the establishment of a Treg-dominant suppressive network; thus, when regulating Tregs, it should be done in conjunction with the extent of viral and antigen control. Two therapeutic sequences can be imagined. In a sequential strategy, viral replication and antigen load are first reduced by nucleoside analogues and, if applicable, emerging antigen-lowering agents; then, Treg-targeted immunomodulation may be added to enhance antiviral immune restoration. Therefore, a safer immunological window may be achieved by reducing the tonic antigenic stimulation before immune intervention. On the other hand, a combined approach would use antiviral and immunomodulatory drugs at the same time to reduce antigen exposure and overcome immune suppression simultaneously. Although theoretically feasible, this way should only be used in a controlled clinical trial environment to avoid immune activation of patients with chronic liver disease causing hepatitis flares or hepatic decompensation.

Development of new HBV-inhibiting drugs will help realise the above combination strategies. Capsid assembly modulators, small interfering RNA (siRNA)-based therapies, antisense oligonucleotides, entry inhibitors, HBsAg release inhibitors and other antigen-reducing agents are being explored to inhibit viral replication, reduce circulating viral proteins and alleviate antigen-driven immune dysfunction. In the phase 2b REEF-1 trial, the siRNA agent JNJ-73763989, in combination with the capsid assembly modulator bersacabavir and nucleoside analogues, reduced HBsAg in patients with chronic HBV infection significantly (74). Although this study did not study Treg-targeted therapy, lowering the antigen load may create a favourable environment for the following immune response. Therefore, future research may explore Treg modulation in conjunction with antigen-lowering combination therapies for patients with persistently low Treg levels and impaired HBV-specific effector functions despite antiviral treatment.

#### 3.6.3 Antigen-specific and liver-targeted Treg modulation

One deficiency of the present Treg-directed approach is a lack of specificity. Widespread depletion of CD25+ cells or systemic checkpoint blockade/non-selective cytokine inhibition can disrupt protective immune tolerance and increase the risk of hepatitis flare, infection or autoimmunity. Specific ways that focus on disease-related Treg populations or limit immunomodulation to the liver may therefore be more feasible for translation. HBV antigen-specific Tregs are therefore the focus of research. HBV core antigen-specific regulatory T cells have been identified in chronic hepatitis B with acute exacerbation (75), and it is now known that some Tregs are associated with virus-specific immune regulation. If specific Treg clones preferentially suppress the antiviral response to HBV, their identification can be achieved by means of MHC class II tetramers, single-cell transcriptomics and TCR sequencing, or paired antigen-reactivity assays, and then selective functional modulation can be carried out. However, these cells are likely to be rare, anatomically compartmentalised, and shaped by the stage of the disease and human leukocyte antigen (HLA) background; thus, their detection and therapeutic targeting are technically challenging.

Cell engineering is also a type of increased specificity. Chimeric antigen receptor regulatory T cells (CAR-Tregs) or TCR-engineered Tregs could, in principle, provide antigen or tissue specificity to adoptively transferred regulatory cells. Such ways may be more suitable for environments that require local immune suppression, such as autoimmune liver disease and transplantation tolerance (71–73). However, in chronic HBV infection, engineered Treg strategies are still mostly theoretical and far from clinical application. At present, their value is mainly heuristic: they show how to address the lack of specificity in systemic Treg modulation eventually. An HBsAg-specific effector TCR strategy has shown technical feasibility in a highly selected HBV-associated HCC case after liver transplantation (79). This report serves only to demonstrate the technical feasibility of HBV antigen-directed cellular engineering; it is not evidence for Treg therapy, cirrhosis treatment, or a foundation for expanding the review to HCC.

Nanoparticle-mediated delivery can also be used to target the liver selectively without cell modification. Liver-targeted nanomedicine platforms have been explored for inflammatory and fibrotic diseases of the liver, and delivery systems aimed at hepatic macrophages, hepatic stellate cells, hepatocytes or liver sinusoidal endothelial cells have supported the feasibility of cell-selective drug delivery in diseased liver tissue (76). Theoretically, such platforms could be employed to deliver HBV antigens, cytokine modulators, small molecules and nucleic-acid-based agents to specific intrahepatic immune or stromal compartments. Therefore, systemic immune perturbation is avoided, and only local modification of the Treg/Th17 axis or Treg-myeloid interactions is permitted. Nanoparticle-mediated Treg modulation has not been studied in the context of HBV-associated cirrhosis, and its safety for patients with portal hypertension, impaired liver function or cirrhosis-associated immune dysfunction is unknown.

Pharmacological stabilisation of FOXP3 expression and Treg lineage identity may be required in the case of Treg instability rather than Treg excess. FOXP3 stability and suppressive function are regulated by epigenetic and post-translational mechanisms, including lysine acetylation. Isoform-selective histone/protein deacetylase modulation has been shown to increase FOXP3 acetylation and Treg suppressive function in transplantation models (77). HBV-associated liver disease is an exception to the above strategies. Restoring the stability of Tregs may be suitable if the inflammatory injury suggests a lack of regulation, but excessive stabilisation of suppressive Tregs could theoretically promote viral persistence.

IL-2 mimetics and other Treg-selective IL-2 pathway agonists can help overcome the narrow therapeutic window of conventional low-dose IL-2 by affecting non-regulatory T cells and Tregs differently. Rezpegaldesleukin, also known as NKTR-358, is a PEGylated IL-2 pathway agonist that can induce stable and selective expansion of Tregs in healthy volunteers and patients with systemic lupus erythematosus (70). Whether this greater selectivity translates into a favorable risk-benefit profile in HBV-associated liver disease remains unknown. Future research will distinguish between injury-dominant states and, on the other hand, chronic high-antigen or immune-tolerant states; in the latter case, additional expansion of suppressive Tregs may inhibit antiviral immune recovery.

Another goal is to recover the spatial context of Treg phenotypes identified by dissociation single-cell analysis. Spatial transcriptomics has resolved injury-associated macrophage and HSC populations and spatially patterned ligand-receptor relationships in fibrotic human liver (80). Complementary MIBI-TOF can quantify dozens of protein markers at subcellular resolution while preserving tissue architecture (81). In combination with scRNA-seq and TCR sequencing, it can be determined whether specific suppressive Treg states are co-localised with scar-associated myeloid cells and activated HSCs in fibrotic septa, portal tracts or regenerative nodules, and whether these niches differ by disease stage or treatment. However, these platforms have not yet defined Treg-myeloid-HSC niches specifically in HBV-associated cirrhosis, and spatial proximity or predicted ligand-receptor pairs cannot establish causal relationships without functional validation.

#### 3.6.4 Implications for the liver transplantation context

A transplantation system shows that Treg modification cannot be divided into just ‘promotion’ or ‘suppression’. Strengthening regulatory immunity in patients with HBV-associated cirrhosis at the end stage of liver disease may help achieve allograft tolerance and reduce the required duration of broad immunosuppression. However, it has not been achieved in practice. A trial of low-dose IL-2 after liver transplantation was planned to expand Tregs and facilitate immunosuppression withdrawal, but it was terminated early because IL-2 failed to expand donor-specific Tregs or promote their hepatic trafficking and was associated with rejection (69). Therefore, the whole-body increase of circulating Tregs alone is not expected to be sufficient to achieve graft tolerance in practice. The potential impact of Treg-directed immunomodulation on post-transplant HBV recurrence also requires specific consideration. Although effective nucleos(t)ide analogue therapy and antiviral prophylaxis have markedly reduced recurrent HBV infection after liver transplantation, excessive enhancement of Treg-mediated suppression could theoretically weaken HBV-specific immune surveillance and favor viral persistence if virological control is incomplete. Conversely, excessive attenuation of Treg activity could increase the risk of graft-directed immune injury and rejection. Therefore, Treg-targeted interventions in HBV-related liver transplant recipients should be evaluated only under effective antiviral prophylaxis and should incorporate serial monitoring of HBV DNA, HBsAg, liver graft function, and HBV-specific antiviral immune competence. At present, the effect of Treg modulation on post-transplant HBV recurrence remains undefined. Patients with a prior or concurrent HCC indication should be analyzed as a separate oncological subgroup: evidence linking intratumoral Treg and PD-L1 enrichment to post-resection recurrence (82) is cited only as a caution against indiscriminate Treg enhancement and does not constitute evidence for Treg therapy in cirrhosis or for transplantation tolerance.

Overall, the clinical development of Treg-targeted therapy in HBV-associated liver disease should move from descriptive immune profiling toward biomarker-defined interventional studies (83). The key question is not whether Tregs should be globally expanded or depleted, but which Treg subset should be modulated, in which anatomical compartment, at which disease stage, and under what antiviral background. A stage-specific, antigen-aware, and biomarker-guided strategy may help convert Treg biology from a marker of immune imbalance into a clinically actionable target for precision immunomodulation.

## 4 Conclusion

In short, Tregs in HBV infection and the development of HBV-associated cirrhosis cannot be regarded as either “increased” or “decreased”; instead, they continuously change in function with changes in the stage of the disease. Tregs limit excessive immune-mediated damage to the body during acute infection. During chronic infection, Tregs suppress HBV-specific antiviral immune responses and may thereby facilitate viral persistence. Progression to liver fibrosis and cirrhosis leads to increased immunosuppression by Tregs, a Treg/Th17 imbalance, and thus promotes the fibrotic microenvironment. Thus, Tregs have dual functions in HBV-related liver disease.

In the future, more research will be conducted on the phenotype, function and microenvironmental interactions of Tregs at different times during disease, rather than solely on quantitative changes. Treg-targeted interventions should also be stage-specific and precise, aim to restore antiviral immunity without worsening hepatic inflammatory injury, and provide new ideas for immunomodulatory therapy of HBV-associated cirrhosis.
